# The Role of Revascularization in Chronic Stable Angina: Do We Have an Answer?

**DOI:** 10.7759/cureus.8210

**Published:** 2020-05-20

**Authors:** Talha Ahmed

**Affiliations:** 1 Internal Medicine, University of Maryland Medical Center, Baltimore, USA

**Keywords:** acute coronary syndrome, percutaneous coronary intervention, coronary artery bypass grafting, chronic stable angina, coronary artery bypass grafting(cabg), optimal medical management

## Abstract

Coronary artery disease is one of the leading causes of death in the United States. The utility of revascularization in patients presenting with acute coronary syndrome is well elucidated, both in reducing death and re-infarction. However, in patients with chronic stable angina, the optimal management strategy is less clear. While medical management with aggressive control of risk factors and lifestyle changes are the cornerstones of treatment, the utility of revascularization in this subset of patients has always been a topic of debate.

## Editorial

Ever since the advent of revascularization strategies, investigators have tried to assess their role in chronic stable angina (CSA) patients. Results from the early studies demonstrated the benefit of coronary artery bypass grafting (CABG) in the high-risk subgroup of CSA patients (those with left main disease, multivessel disease, left ventricular dysfunction, and proximal left anterior descending [LAD] artery). This advantage was seen on top of optimal medical management (OMT). However, the results of these studies performed between the 1970s and 1990s has been challenged in the current era [1]. The reason is twofold: one being the advancement in medical management, while the other being the adoption of better revascularization strategies. 

In 2007, results from the Clinical Outcomes Utilizing Revascularization and Aggressive Drug Evaluation (COURAGE) trial demonstrated that adding a catheter-based revascularization to OMT did not affect outcomes of cardiovascular death or myocardial infarction (MI) in CSA patients. The results of this study were further supported by the Bypass Angioplasty Revascularization Investigation Diabetes (BARI-D) trial. In this study, among diabetics, revascularization did not reduce the risk of major adverse cardiovascular events (MACE), cardiovascular death, and all-cause mortality. In 2010, results from a meta-analysis of 15 randomized studies looking at the cost-effectiveness of revascularization compared to medical management revealed a 10 times higher cost of CABG than OMT. The cost of percutaneous coronary intervention (PCI) lied in between this spectrum. In another interesting head to head comparison of the two revascularization strategies, the SYNergy between percutaneous coronary intervention with TAXus and cardiac surgery (SYNTAX) trial showed a high incidence of MACE in the PCI group than in the CABG group. These results were particularly true for patients with high syntax scores at one, three, and five-year follow-ups. These results were supported later by the Future REvascularization Evaluation in patients with Diabetes Mellitus: Optimal management of Multivessel disease (FREEDOM) trial and BARI trial that demonstrated the superiority of CABG versus PCI driven primarily by an advantage seen in patients with high-risk lesions (left main disease, multivessel disease, or proximal LAD artery disease). As a result of these studies, the rates of PCI performed in CSA patients decreased by 61% from 2006 to 2011 [2]. 

In 2011, the Surgical Treatment for IsChemic Heart failure (STICH) trial evaluated the role of CABG as compared to OMT in CSA patients with left ventricular dysfunction. Although the two groups did not differ in primary outcomes, the secondary outcomes of the rate of death due to cardiovascular (CV) causes and hospitalization for CV causes were low in the CABG arm. These results were interesting as investigators were able to demonstrate the fact that treating the ischemia based on imaging, did not change outcomes in CSA patients [2]. In the Fractional flow reserve versus Angiography for Multivessel Evaluation 2 (FAME-2) trial, a fractional flow reserve (FFR)-based approach to revascularization showed no difference in primary outcomes of death and MI in the FFR-based PCI group and the OMT group. These results are not surprising as earlier studies have shown that the lesions which are responsible for acute coronary syndrome (ACS) in patients with CSA are not necessarily the ones that are more hemodynamically limiting. Any unstable plaque with a thin coat and lipid-laden core, regardless of size, can erode or rupture leading to ACS. This finding was validated by the Providing Regional Observations to Study Predictors of Events in the Coronary Tree (PROSPECT) trial in 2012. This trial enrolled patients with ACS who had extensive three-vessel imaging at their index hospitalization. Later, when these patients presented with ACS at follow-up, more than half of these lesions at subsequent presentations were not significant at all at the index presentation [2]. In summary, to the investigators' dismay, neither a viability study driven approach nor an FFR-based significant lesion strategy to revascularization seemed to improve outcomes in CSA when added to OMT.

According to the American College of Cardiology/American Heart Association (ACC/AHA) 2014 guidelines, CABG is indicated for the patients with CSA having left main disease, three and two-vessel disease, and proximal LAD artery disease (Class of recommendation I and level of evidence B) for improving survival. Similarly, CABG is performed to improve angina in patients with disabling symptoms (Class of recommendation I and level of evidence A). The European guidelines are more liberal in performing revascularization and do not favor CABG over PCI in patients with left main disease, multivessel disease, proximal LAD disease, a large area of ischemia (more than 10%), and single remaining vessel. Moreover, revascularization is recommended not only to improve survival but also to improve the quality of life in the European guidelines for all these indications [3,4]. Another interesting and yet negative study was a sham-controlled study of revascularization versus medical management in CSA patients at a six-week follow-up using the Seattle Angina Questionnaire (SAQ). The results of this study demonstrated no benefit of revascularization with PCI in addition to OMT in patients with CSA [5].

The International Study of Comparative Health Effectiveness with Medical and Invasive Approaches (ISCHEMIA) trial is the largest ever study performed to date in an attempt to solve this dilemma and was reported recently in 2020 [5]. This is an International, randomized control, open-label study. The largest recruitment was from India. In this trial, 5,179 patients were randomized to OMT arm versus OMT plus revascularization arm (PCI or CABG as determined by the performing center). Patients with moderate to severe ischemia on perfusion imaging or exercise stress testing were included. The trial excluded patients with left main and non-obstructive disease (determined by angiography or cardiac computed tomographic angiography after randomization), advanced heart failure patients, and those with disabling angina symptoms. After a median follow-up period of 3.3 years, no difference in hard outcomes (a primary composite outcome of cardiovascular death, MI, resuscitated cardiac arrest, or hospitalization for unstable angina or heart failure) was noticed. The extent of ischemia or the severity of ischemia did not affect the results of this study. A modest improvement of quality of life was reported in OMT plus revascularization arm but as a counterargument, only a small percentage of patients in this trial had limiting angina, to begin with. 

The results of this study would make one think: Did we get our answer? Does revascularization actually does not work in CSA patients? There, unfortunately, is still a lot that needs to be taken into account. The results of this trial are not generalizable to all the CSA patients (excluded patients with left main coronary artery disease as well as non-obstructive coronary artery disease). As compared to Europe, cardiologists in the United States were already performing revascularization in the selected group of these patients and the guidelines also demonstrate the same. The event rate was low in this study in either arm. One can argue that if the follow-up interval was stretched to 10 or 15 years (which obviously is not possible and is a limitation of randomized studies), the results may have favored one arm over the other. The medical management was not optimized in a good number of patients in either arm. This would not affect the comparability of outcomes but certainly point towards an easier approach to the problem. Can a focused approach including close follow-ups, introducing the concept of 'angina clinics' to monitor these patients and active utilization of behavioral therapies to adopt healthy lifestyle measures change outcomes in CSA patients? One cannot answer that question with certainty, particularly when not supported by the results of a study but this definitively is an area to improve.

To conclude, as with other studies performed earlier, the results of the ISCHEMIA trial do have limitations and should be carefully extrapolated to the clinical situations faced in real-world practice. An individualized approach to revascularization and meticulous attention to medical and lifestyle management in both primary care visits and cardiology outpatient visits can help mitigate the burden of this disease and improve patient outcomes. 
